# Predictors of mortality among elderly dependent home care patients

**DOI:** 10.1186/1472-6963-13-316

**Published:** 2013-08-15

**Authors:** Joan Gené Badia, Alícia Borràs Santos, Joan Carles Contel Segura, Carlos Ascaso Terén, Laura Corredoira González, Ester Limón Ramírez, Pedro Gallo de Puelles

**Affiliations:** 1 CAPSE Consorci d’Atenció Primària de Salut de l’Eixample, c/ Roselló 161, Barcelona 08036, Spain; 2 Institut Català de la Salut, Barcelona, España; 3 Departament de Salut Pública, Universitat de Barcelona, Barcelona, España; 4 Institut de Recerca Jordi Gol i Gurina, Barcelona, España; 5 Departament de Sociologia i Anàlisi de les Organitzacion, Universitat de Barcelona, Facultat Economia i Empresa, Barcelona, España

**Keywords:** Primary care, Home care, Elderly patients, Dependency, Mortality

## Abstract

**Background:**

The purpose of this study is to identify which variables –among those commonly available and used in the primary care setting– best predict mortality in a cohort of elderly dependent patients living at home (EDPLH) that were included in a home care program provided by Primary Care Teams (PCT). Additionally, we explored the risk of death among a sub-group of these patients that were admitted to hospital the year before they entered the home care program.

**Methods:**

A one-year longitudinal cohort study of a sample of EDPLH patients included in a home care programme provided by 72 PCTs. Variables collected from each individual patient included health and social status, carer’s characteristics, carer’s burden of care, health and social services received.

**Results:**

1,001 patients completed the study (91.5%), 226 were admitted to hospital the year before inclusion. 290 (28.9%) died during the one-year follow-up period. In the logistic regression analysis women show a lower risk of death [OR= 0.67 (0.50-0.91)]. The risk of death increases with comorbidity [Charlson index OR= 1.14 (1,06-1.23)], the number of previous hospital admissions [OR= 1,16 (1.03-1.33)], and with the degree of pressure ulcers [ulcers degree 1–2 OR = 2.94 (1.92-4.52); ulcers degree 3–4 OR = 4.45 (1.90-10.92)]. The logistic predictive model of mortality for patients previously admitted to hospital identified male sex, comorbidity, degree of pressure ulcers, and having received home care rehabilitation as independent variables that predict death.

**Conclusions:**

Comorbidity, hospital admissions and pressure ulcers predict mortality in the following year in EDPLH patients. The subgroup of patients that entered home care programs with a previous record of hospital admission and a high score in our predictive model might be considered as candidates for palliative care.

## Background

A major challenge for family doctors today is to provide appropriate care for the growing elderly dependent population living at home (EDPLH) that are unable to pay a visit to existing health centre facilities. EDPLH patients represent approximately 10% of the population over 65 years of age [1], and would be labelled as frail patients under a geriatric definition [2]. This group of patients is traditionally included in home care programmes run by primary care nurses and family doctors.

There is a reported increased risk of death following hospital admission in these patients, and primary care interventions that provide integrated social and health care services to them have demonstrated a reduction in hospital admission rates [3,4]. In this respect, previous published studies have identified comorbidity, discontinuity of care, demand and social problems as major causes of hospital admissions among EDPLH patients benefitting from home care programmes [5,6]. Further, it is known that pressure ulcers are a major predictor of death in patients living at home or in nursing homes. Existing studies have shown that pressure ulcers double the risk of death for these patients during the subsequent year [7,8].

Comorbidity, dependency and poor self-perceived physical and mental health status have also been identified as independent predictors of mortality among the elderly population [9-15]. Despite comorbidity being associated to an increased risk of death, some studies have argued that it is the combination of specific pathologies which explains this risk [16,17]. These studies, however, refer to community dwelling old patients in general and not to EDPLH patients in particular.

The purpose of this study is to identify which variables –among those commonly available and used in the primary care setting- best predict mortality in a cohort of EDPLH patients benefitting from home care programmes in primary care. Our interest is therefore to explore how patient’s characteristics, carer’s and services dimensions relate to the probability of dying, both for all EDPLH patients included in the study and for a subgroup of patients that were admitted into hospital care the year before inclusion in the home care program.

## Methods

This is a longitudinal one-year follow-up study of a cohort of elderly patients living at home (EDPLH). The methodology of the study has been published elsewhere [6,18]. This study was conducted in Catalonia, a region in Spain with a National Health Service that provides health care to every citizen free of charge at the point of delivery. Primary care in the region is provided by 340 Primary Care Teams (PCT), with the participation of family doctors and community nurses, each of them covering an area ranging from 5 to 25 thousand inhabitants. Each individual PCT delivers a home care programme addressing the needs of patients who cannot pay a regular visit to a primary care centre. This is done in addition to the regular services provided within primary care facilities, which consists of basic medical assistance and nursing care as well as an emergency service, both at the primary care centre and at the patients’ home. In addition, other centralised 24h emergency services are available, activated by patients’ telephone calls.

The research protocol of this study was approved by the Ethics and Research Committee in the Jordi Gol i Gurina Primary Care Research Institute.

Criteria for inclusion required patients to be non-institutionalised, chronically ill [19], 65 years of age and over, who were unable to autonomously seek care in a primary health care centre. Patients selected for the study were already included in a home care programme. The recruitment period was between the 1st July and the 31st December 2005. Patients who had been admitted to hospital care before the baseline assessment were considered as a subgroup and analysed separately.

Patient exclusion criteria included refusal to participate, transitory patients (followed by the primary care team for less than 9 months/year), patients with a life expectancy below 4 months, patients receiving transitory post-surgical care, and when both the patient and the carer had been diagnosed with dementia.

Informal carers are defined in the study as those looking after the basic needs of the patient, receiving no specific remuneration for that task and having a close relationship (next-to-kin or friend) with the patient.

### Sampling

Regarding patient recruitment, each of the 378 primary health care professionals who agreed to cooperate in the study selected, on average, 3 patients fulfilling the inclusion criteria. Participating professionals work throughout the Catalan territory, including rural and urban areas. They collaborated in the study on a voluntary basis and no randomisation was performed. They were asked to include in the study at least the first three patients that were found eligible under the agreed inclusion and exclusion criteria. Each of these professionals acted as collaborative researcher and a member of the research team trained them in their tasks. Health professionals were responsible for the administration of the questionnaires used in this study and the gathering of the necessary patient data from clinical visits, clinical records and reports from other health care providers.

### Data collection

The following data were collected from each patient at baseline and after one year follow-up: patient clinical characteristics, comorbidity level (Charlson test) [20], functional status (Barthel test) [21], cognitive status (Pfeiffer test) [22], existence of decubitus ulcers, risk of appearance of ulcers (Braden test) [23], subjective health status (SF-12) [24], social risk (Gijon Test) [25] and carer burden (Zarit test) [26]. All the questionnaires mentioned above have been validated for use in a Spanish context.

In addition we looked into variables that measured the utilisation of health and social services including hospital admissions (defined as the patient remaining in acute hospital care for over 24 h), emergency room visits, home emergency visits data, primary care and community services activity data. The utilisation of social services included home help, tele-assistance, meals on wheels, volunteer care, social work visits, day centre and nursing homes, among others. These variables were collected for all patients one year before the baseline assessment and during the one year follow up period.

The utilisation of health and social services the year before the baseline assessment was extracted from patient’s clinical records. Collaborative researchers in the study also collected the same utilisation variables prospectively during follow up.

Researchers participating in the study received training for data collection standardisation and collected data were analysed centrally. A continuous data quality assurance process was in place in order to minimize errors and information losses. A research team member audited and validated 10% of the data gathered by collaborative researchers from patients’ original clinical records. The Kappa index and interclass correlation index were used to test the concordance among registers [6]. The results showed an adequate concordance.

### Statistical analysis

A descriptive analysis is provided including average and standard deviation for all continuous variables. We analysed baseline differences between individuals who died and those who did not die during the study period by means of the Mann–Whitney-Wilcoxon non-parametric test. Categorical variables were analysed using the chi-square test and Fisher’s exact test. We agreed on a p-value significance level of p < 0.05.

Following a bivariate analysis we included significant variables in a logistic regression model, in which the dichotomous dependent variable was vital status. We used stepwise techniques for inclusion and exclusion of variables in the model, testing the significance of each individual variable by means of a deviance analysis. We ran two separate analyses, one including all patients in the sample, and a second analyses including only patients with an informal carer. Accordingly, carer-related variables were excluded from the first analysis since not all patients had an informal carer. The statistical results are expressed in terms of odds ratios (OR) and 95% confidence intervals (CI). The statistical package used was R [27].

We fitted a second logistic regression model in order to identify those variables that best explained the event of death among those patients that were hospitalised the year before entering the home care program. The resulting predictive model shows the probability of death among home care patients that were hospitalised before entering the program, in terms of patient’s characteristics, carer’s and service variables. We used the Hosmer-Lemeshow test to report the goodness of fit and ROC curve analysis to assess the discriminatory power of the model.

## Results

A total of 1,093 EDPLH patients were included in the study. Patients that completed the study (1,001) had a mean age of 83.7 years (±6.8 sd.), 66.8% were female (729). 290 patients died during the one year follow-up (28.9%).

As reported in Table 1, patients lost to follow-up (92) had similar characteristics to patients who were followed up (1,001), with the exception of the former receiving proportionally more rehabilitation at home and having a higher average number of days temporarily admitted to nursing homes.

**Table 1:** Differences between patients lost to follow-up and followed-up patients

	**Patients lost to follow-up (n=92)**		**Patients followed up (n=1001)**		***p***
	**N**	**%**	**N**	**%**	
**Rehabilitation at home**	16	17.4	92	9.2	<0.05
	**Mean**	**SD**	**Mean**	**SD**	
**Temporary admissions in nursing homes (number of days)**	5.0	22.2	1.33	8.35	<0.05

Regarding personal characteristics, health status and life-styles of patients included in the study, Table 2 shows there are some significant differences between those who died and those who survived until the end of the study. Indeed, female gender is a relevant variable in the study associated to survival both in the whole population as well as in the hospitalized subgroup. Similarly, individuals who survived in both groups showed lower comorbidity, higher levels of autonomy, less risk of pressure ulcers and lower prevalence of pressure ulcers. In the entire sample –in contrast to what was observed in the sub-group of patients admitted to hospital–, survival was associated to less cognitive impairment and higher score in physical and mental self-perceived health status.

**Table 2:** Patients’ characteristics, health status and lifestyles

	***Total population***			***Patients admitted to hospital the year before entering the home care program***		
	**Survived (n=711)**	**Died (n=290)**	***p***	**Survived (n=138)**	**Died (n=88)**	***p***
**PERSONAL CHARACTERISTICS**						
**Gender, female, (%)**	497 (69.9)	164 (56.5)	<0.001	85 (61.6)	35 (39.8)	<0.001
**Age (years), mean +/− sd**	84.4 ± 6.7	84.43 ± 6.7	NS	83.36 ± 6.85	83.77 ± 6.26	NS
**Smoker, Yes (%)**	24 (3.4)	6 (2.1)	NS	4 (2.9)	2 (2.3)	1
**Alcohol consumption**						
Risk (%)	6 (0.8)	3 (1.0)	NS	0 (0.0)	2 (2.3)	NS
Moderate (%)	47 (6.6)	17 (5.9)		9 (6.5)	5 (5.7)	
No (%)	655 (92.1)	270 (93.1)		129 (93.5)	81 (92.0)	
**HEALTH STATUS**						
**Comorbidity, mean +/− sd(Charlson Index)**	2.1 ± 1.9	2.9 ± 2.0	<0.001	2.64 ± 2.0	3.81 ± 2.2	<0.001
**Autonomy, mean +/− sd (Barthel Index)**	64.7 ± 25.9	53.4 ± 31.4	<0.001	62.9 ± 25.5	51.9 ± 32.5	<0.05
**Cognitive status, mean +/− sd (Pfeiffer Test)**	3.2 ± 3.0	4.2 ± 3.6	<0.001	3.4 ± 2.9	4.1 ± 3.8	NS
**Pressure ulcers risk, mean +/− sd (Braden Test)**	18.9 ± 3.2	18.1 ± 3.6	<0.001	18.64 ± 3.3	17.5 ± 3.7	< 0.05
**Presence of pressure ulcers**						
No (%)	648 (91.1)	219 (75.5)	<0.001	124 (89.9)	64 (72.7)	<0.05
Stage 1–2 (%)	51 (7.2)	57 (19.7)		9 (6.5)	18 (20.5)	
Stage 3–4 (%)	9 (1.3)	14 (4.9)		5 (3.6)	6 (6.8)	
**Health-related-quality of life (SF-12)**						
Physical Composite Score, mean +/− sd	31.5 ± 8.0	29.9 ± 7.2	<0.05	30.5 ± 8.0	28.6 ± 7.7	NS
Mental Composite Score, mean +/− sd	41.2 ± 12.3	38.8 ± 12.7	<0.05	39.8 ± 12.6	38.1 ± 12.1	NS
**Social risk, mean +/− sd (Gijón Test)**	10.8 ± 3.0	10.7 ± 3.0	NS	10.6 ± 2.8	10.9 ± 3.0	NS

NS= p>0.05.

Sd= Standard Deviation.

Score range of the scales: Charlson 0–5: Barthel: 0–100; Zarit: 0–110; Pfeiffer : 0–10; Braden: 0 – 23; Gijon : 0 – 25; Physical Composite and Mental Composite of SF 12 : 0–100.

Furthermore, patients who died during follow-up received a higher proportion of informal care, largely provided by women carers, and these informal carers reported a higher burden of care as measured by the Zarit test (see Table 3). In contrast, informal care was not found to be a significant variable in explaining survival among patients that were hospitalised the year before they entered the home care program.

**Table 3:** Informal carer characteristics and overburden at the basal assessment

	***Total population***			***Patients admitted to hospital the year before entering the home care program***		
	**Survived (n=711)**	**Died (n=290)**	***p***	**Survived (n=138)**	**Died (n=88)**	***p***
**With informal carer N (%)**	571 (80.3)	250 (86.2)	<0.05	115 (83.3)	80 (90.9)	NS
**Carer age, mean +/− sd (N= 821)**	61.9 ± 13.9	63.5 ± 14.31	NS	63.8 ± 14.3	63.7 ± 12.5	NS
**Carer gender, female N (%) (N= 821)**	444 (62.5)	208 (71.7)	<0.05	93 (67.4)	69 (78.4)	NS
**Zarit Test, mean +/− sd (N= 821)**	50.2 ± 15.9	54.7 ± 17.2	<0.001	51,1 ± 17.2	56.4 ± 17.2	NS

N= number; sd =Standard Deviation; NS= p>0.05.

With respect to health and social services utilisation (Tables 4 and 5), patients dying during the follow-up year received largely the same amount of services in the year before their death as those who survived, with the exception of a lower use of tele-assistance services (8.3% vs. 13.6%) and hospital at home services (1.4% vs. 1.6%), and a higher use of health centre emergency services (17.2% vs. 10.3%), emergency community services (24.8%, 15.3%), and in-patient care (1.6% vs. 0.9%). In the sub-group of 226 patients admitted into hospital before entering the home care program, no differences were found in health and social services utilisation (Tables 4 and 5) except for a higher use of centralized after-hours emergency community services among patients who died.

**Table 4:** Services received the year before the basal assessment

	***Total population (N=1,001)***					***Patients admitted to hospital the year before entering the home care program (N=226)***				
	**Survived (n=711)**		**Died (n=290)**		***p***	**Survived (n=138)**		**Died (n=88)**		***p***
	**N**	**%**	**N**	**%**		**N**	**%**	**N**	**%**	
**Home help (formal carer)**	286	40.2	104	35.9	NS	55	39.9	31	35.6	NS
**Teleassistance**	97	13.6	24	8.3	<0.05	21	15.2	7	8	NS
**Meals on wheels**	8	1.3	1	0.4	NS	2	1.4	1	1.1	1
**Volunteers**	17	2.4	4	1.4	NS	3	2.2	0	0	NS
**After-hours emergency centralized community services**	109	15.3	72	24.8	<0.001	49	35.5	44	50	<0.05
**Hospital at Home services**	11	1.6	4	1.4	<0.001	5	3.6	2	2.3	NS
**Health center emergency services**	73	10.3	50	17.2	<0.001	21	15.3	22	25	NS
**Rehabilitation at Home**	66	9.3	20	6.9	NS	25	18.1	9	10.2	NS
**Palliative home care services**	22	3.0	15	5.2	NS	9	6.5	2	2.3	NS
**Private health services**	61	8.6	19	6.6	NS	12	8.7	5	5.7	NS
**Attendance at the social services day center**	11	1.6	6	2.1	NS	4	2.9	1	1.1	NS
**Other social services**	19	2.7	14	4.9	NS	5	3.6	5	5.7	NS
**Day Hospital**	16	2.3	8	2.8	NS	7	5.1	6	6.8	NS

N= number; NS= p>0.05.

**Table 5:** Type of services received by EDPLH patients during the one year follow-up period

	**Survived (n=711)**		**Died (n=290)**		***p***	**Survived (n=138)**		**Died (n=88)**		***p***
	**Mean**	**±SD**	**Mean**	**±SD**		**Mean**	**±SD**	**Mean**	**±SD**	
**Social worker visits**	0.4	1.0	0.4	1.0	NS	0.6	1.3	0.5	1.4	NS
**Hours/week of home help**	18.5	41.8	21.6	49.0	NS	23.1	47.7	24.2	52.6	NS
**Family doctor visits**	4.0	5.1	3.8	5.4	NS	4.7	3.9	5.4	4.6	NS
**Nurse visits**	7.9	7.6	8.7	14.6	NS	10.5	11.3	10.8	8.6	NS
**Number of days per patient as temporal admissions in a nursing home**	2.4	13.7	2.08	10.5	NS	5.3	20.9	5.6	17.1	NS
**Hospital emergency services visits**	0.64	1.3	0.8	1.6	NS	1.5	1.8	1.7	2.4	NS
**Number of Hospital admissions (more 24 Hours)**	0.31	0.9	0.6	1.6	<0.0001	1.6	1.3	1.9	2.3	NS

N= number; sd =Standard Deviation; NS= p>0.05.

Variables found to be independently associated to the risk of dying during follow-up in this EDPLH group (N=1,001) were male gender, comorbidity (as measured by the Charlson test), the number of hospital admissions the year before, and both the existence of and degree of pressure ulcers (see Table 6). When considering the EDPLH group with an informal carer (N=821) we found the same variables associated to risk of dying as in the total sample with the exception of gender, and two additional variables, namely self assessed health status (as measured by SF-12) and burden on the carer (as measured by the Zarit test) (see Table 7).

**Table 6:** Independent risks of dying during the following year (N=1,001) (logistic regression analysis)

**Variable**	***Odds ratio (95% IC)***	***p***
Constant	0.31	0.000
Gender, Female	0.68 (0.50 - 0.92)	0.014
Comorbility (Charlson Index)	1.14 (1.06 - 1.23)	0.000
Number of hospital admissions (>24 hours)	1.17 (1.01 - 1.35)	0.038
Pressure ulcers stage 1-2	3.00 (1.94 - 4.65)	0.000
Pressure ulcers stage 3-4	4.33 (1.81 - 10.4)	0.001
Health-related-quality of life (SF-12)	1.03 (0.98 - 1.08)	0.272
Social risk (Gijón Test)	0.99 (0.95 - 1.05)	0.840
Hospital at home service	0.82 (0.60 - 1.11)	0.200
Teleassistance	0.69 (0.42 - 1.15)	0.152
Number of hospital emergency visits during last year	1.00 (0.89 - 1.12)	0.984

CI= Confidence Interval.

**Table 7:** Independent risks of dying during the following year in population with informal carer (N = 821) (logistic regression analysis)

**Variable**	***Odds ratio (95% IC)***	***p***
Constant	0.15	0.000
Gender, Female	0.79 (0.56 - 1.12)	0.189
Zarit Test	1.01 (1.00 - 1.02)	0.012
Comorbility (Charlson Index)	1.17 (1.08 - 1.27)	0.000
Number of hospital admissions (>24 hours)	1.18 (1.01 - 1.37)	0.035
Pressure ulcers stage 1-2	2.66 (1.65 - 4.28)	0.000
Pressure ulcers stage 3-4	4.33 (1.63 - 11.5)	0.003
Health-Related-Quality of life (SF-12)	1.58 (1.06 - 2.36)	0.025
Social risk (Gijón Test)	1.00 (0.94 - 1.05)	0.874
Hospital at home service	0.76 (0.53 - 1.09)	0.142
Teleassistance	0.94 (0.51 - 1.71)	0.835
Number of hospital emergency visits during last year	0.97 (0.86 - 1.10)	0.639

CI= Confidence Interval.

Variables that predict the risk of dying among those patients that were hospitalised before entering the home care program were male gender, comorbidity (as measured by the Charlson test), having received home care rehabilitation, and both the existence and degree of pressure ulcers. The resulting predictive model is represented by the formula below. The Homer-Lemeshow goodness of fit test is p=0.4837. The probability of dying during the next year can be calculated by the following equation:

$$\begin{array}{ll} \;\text{Probability of death}= & -3.423+1.951\\ & \times \left(\text{if patient is a male}\right)+0,449\\ & *\left(\text{Charlson index value}\right)-0.404\\ & *(\text{Charlson index value if patient}\\ & \;\text{is male sex})+1.180\\ & \times (\text{if the patient received home}\\ & \;\text{care rehabilitation})+1.444\\ & \times (\text{if}\;1^{\mathrm{st}}-2^{\mathrm{nd}}\text{degree pressure ulcers}\\ & \;\text{are present})+1.594\\ & \times (\text{if}\;3^{\mathrm{rd}}-4^{\mathrm{th}}\text{degree pressure ulcers}\\ & \;\text{are present}). \end{array}$$

**Figure 1:**
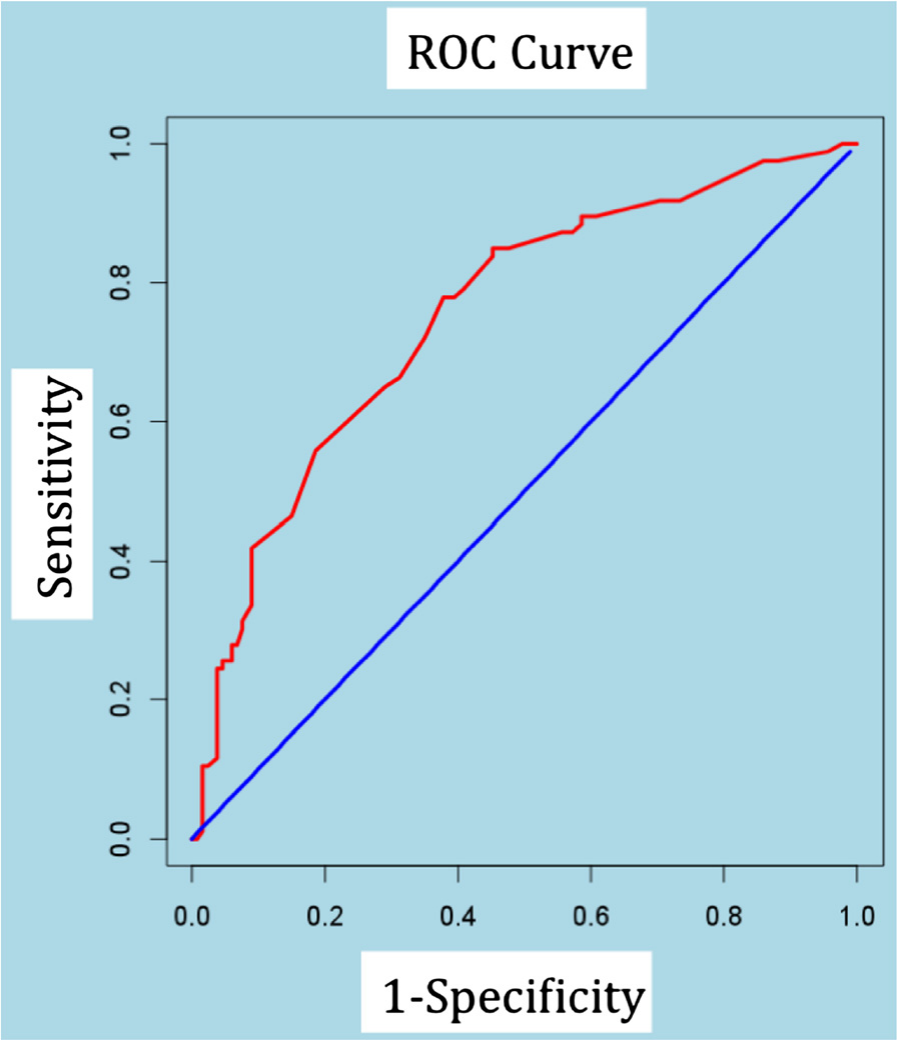
Predictive model of death in EDPLH patients admitted to hospital the year before entering the home care program: ROC curve.

The area below the ROC curve is 0.754 (95%CI=0.689-0.820) (see Figure 1). The sensitivity and specificity values are 65.12% and 71.11%, respectively. True positive predictive value is 0.59 and true negative predictive value is 0.76.

## Discussion

EDPLH patients constitute a frail population with a high risk of dying. Our study shows that approximately a quarter of these patients died during the one-year follow-up period. Variables that independently predict death are found to be male gender, comorbidity, the existence of pressure ulcers and the number of hospital admissions the year before the baseline assessment. Among patients with an informal carer, those EDPLH patients with worse perceived and objective health status who are looked after by heavily burdened informal carers have a greater probability of dying during the following year. Since hospital admission was identified as a risk factor for death, we went further and built a predictive model of death for that sub-group of patients. This model yielded a formula of an acceptable discriminative power that includes sex (male), comorbidity (as measured by the Charlson index), degree of pressure ulcers, and having received home care rehabilitation. The formula also accounts for the fact that the Charlson index predicts a higher risk of death among women that men. This is a simple formula, that can be easily calculated by the doctor at the bed-side when deciding which would be the best therapeutical path to follow.

The main limitation of this research is that the sample of patients included in the study is not representative of all patients visited by primary care teams, since it was not a randomised sample. However, the sample was large enough to fulfil the objectives of the study. In addition, as patients who were lost to follow-up were probably sicker, this might imply that some of the odds ratios obtained in the study are under-estimates.

Variables found as significant predictors of mortality in EDPLH patients might be associated to other variables such as malnutrition, frailty syndrome and severity of comorbidity, that were not collected in this study.

Contrary to other published research on very elderly populations [28], in the present study physical functionality itself was not associated to mortality. We understand that physical functionality is somewhat hidden in our study since patients included are all home care patients with reduced autonomy, unable to move out of their homes and showing a low Barthel test score.

Previous research with the same cohort has found that hospital admissions are related to lack of coordination among services as well as demand induced by family relatives and patients themselves [6].

Published studies have found that admission to hospital is not always to the benefit of these EDPLH patients because admission could aggravate the pre-existing pathological status of these patients [29-31]. It is thus of paramount importance to identify patients at a high risk of dying during hospitalisation before they are actually admitted. Some predictive models of death for community dwelling elderly patients have been published, that use either self-reported data [32] or clinical data [11,17]. General practitioners and emergency doctors could routinely use these instruments in order to avoid costly [33] and lethal hospitalisation. Family relatives should be informed of the benefits of maintaining these patients at home during their acute episodes [34].

We have contributed to this literature by arguing that the risk of dying does not disappear after hospitalization but prevails thereafter. It is therefore of utmost importance to take the patient’s record of previous hospital utilisation into account when entering a given home care program. Our predictive formula allows for the identification of those patients with a greater probability of a reduced life expectancy (below one year). It could therefore be of assistance in selecting those patients who would benefit from palliative care.

It is worth noticing that none of the variables related to the utilisation of primary health care services during the previous year are related to better survival. On the contrary, the number of hospital admissions increases the risk of death in this EDPLH population. Further, primary care services are bound to have no differential effect on the risk of death since they are available to all patients in largely the same regime. The few published studies looking at the connection between home care services and mortality, have found no relationship [17]. Other studies report improvement in survival as a consequence of providing housekeeping and personal assistance services [35,36], or as a result of these frail patients visiting a day centre [37]. Nursing and medical home care services seem to have little effect if not accompanied by the provision of community social services [38].

In the light of the above findings we would recommend future research into management policies and practices addressing this group of patients. We encourage research on the effectiveness of preventive measures among EDPLH patients in three areas. First, to assess whether a more adequate detection, assessment and treatment of comorbidity would improve survival. Second, to investigate the effectiveness of pressure ulcer prevention actions such as informing carers and family relatives about adequate diet and postural management strategies, and counselling health care services on the public provision of specific mattresses for these patients. Finally, we suggest further research on management strategies for a subgroup of patients that could benefit from a more end-of-life oriented care in their home setting without hospitalisation. This might avoid unnecessary direct and indirect costs to the health care system and to the family. Information systems already in place could serve the purpose of identifying these high-risk patients, allowing for appropriate and timely professional action.

## Conclusions

Family doctors and community nurses could play an active and central role in the management of EDPLH patients using mortality risks assessment in the management of each individual patient. The predictive model presented here could help doctors make decisions on therapeutic alternatives for these EDPLH. This would improve the quality of existing home care programs. High-risk patients could benefit greatly from a more end-of-life oriented care in their home.

## Abbreviations

EDPLH: Elderly dependent patients living at home; PCT: Primary care teams; SF-12: The short form (12) Health Survey is a survey of patient health; OR: Odds ratio; CI: Confidence interval.

## Competing interests

The authors declare that they have no competing interests.

## Authors’ contributions

JGB, ABS and EL participated in the study design, data acquisition, analysis and interpretation, and drafted the manuscript. JCCS, CAT, LCG and PGP were involved in the study design, analysis and interpretation, and drafted the manuscript. The HC>65 Research Team participated in data acquisition. All authors read and approved the final version of the manuscript.

## Pre-publication history

The pre-publication history for this paper can be accessed here:

http://www.biomedcentral.com/1472-6963/13/316/prepub

## HC>65 Research Team

The full list of participants has been published elsewhere: Gené J et al. Los problemas de salud también explican la utilización de servicios sociales en atención domiciliaria Aten Primaria. 2009 Feb; 41(2):91–101.
